# A phase I clinical study of vaccination of melanoma patients with dendritic cells loaded with allogeneic apoptotic/necrotic melanoma cells. Analysis of toxicity and immune response to the vaccine and of IL-10 -1082 promoter genotype as predictor of disease progression

**DOI:** 10.1186/1479-5876-6-6

**Published:** 2008-01-25

**Authors:** Erika M von Euw, María M Barrio, David Furman, Estrella M Levy, Michele Bianchini, Isabelle Peguillet, Olivier Lantz, Alejandra Vellice, Abraham Kohan, Matías Chacón, Cassian Yee, Rosa Wainstok, José Mordoh

**Affiliations:** 1 Centro de Investigaciones Oncológicas FUCA, Cramer 1180, (1426) Buenos Aires, Argentina; 2 Laboratorio de Cancerología, Fundación Instituto Leloir, IIBA -CONICET, Patricias Argentinas 1405, Buenos Aires, Argentina; 3 Laboratoire d'Immunologie, Institut Curie, 26 rue d'Ulm 75005 Paris, France; 4 Unité Inserm 653, Institut Curie, 26 rue d'Ulm 75005 Paris, France; 5 Instituto Médico Alexander Fleming, Cramer 1180, 1426 Buenos Aires, Argentina; 6 Fred Hutchinson Cancer Research Centre, Clinical Research Division, 1100 Fairview Avenue N., D3-100, Seattle, Washington 98109-1024, USA; 7 Departamento Química Biológica. Facultad de Ciencias Exactas y Naturales, Universidad de Buenos Aires, Buenos Aires, Argentina

## Abstract

**Background:**

Sixteen melanoma patients (1 stage IIC, 8 stage III, and 7 stage IV) were treated in a Phase I study with a vaccine (DC/Apo-Nec) composed of autologous dendritic cells (DCs) loaded with a mixture of apoptotic/necrotic allogeneic melanoma cell lines (Apo-Nec), to evaluate toxicity and immune responses. Also, IL-10 1082 genotype was analyzed in an effort to predict disease progression.

**Methods:**

PBMC were obtained after leukapheresis and DCs were generated from monocytes cultured in the presence of GM-CSF and IL-4 in serum-free medium. Immature DCs were loaded with gamma-irradiated Apo-Nec cells and injected id without adjuvant. Cohorts of four patients were given four vaccines each with 5, 10, 15, or 20 × 10^6 ^DC/Apo-Nec cell per vaccine, two weeks apart. Immune responses were measured by ELISpot and tetramer analysis. Il-10 genotype was measured by PCR and corroborated by IL-10 production by stimulated PBMC.

**Results:**

Immature DCs efficiently phagocytosed melanoma Apo-Nec cells and matured after phagocytosis as evidenced by increased expression of CD83, CD80, CD86, HLA class I and II, and 75.2 ± 16% reduction in Dextran-FITC endocytosis. CCR7 was also up-regulated upon Apo-Nec uptake in DCs from all patients, and accordingly DC/Apo-Nec cells were able to migrate *in vitro *toward MIP-3 beta. The vaccine was well tolerated in all patients. The DTH score increased significantly in all patients after the first vaccination (Mann-Whitney Test, p < 0.05). The presence of CD8^+^T lymphocytes specific to gp100 and Melan A/MART-1 Ags was determined by ELISpot and tetramer analysis in five HLA-A*0201 patients before and after vaccination; one patient had stable elevated levels before and after vaccination; two increased their CD8 + levels, one had stable moderate and one had negligible levels. The analysis of IL-10 promoter -1082 polymorphism in the sixteen patients showed a positive correlation between AA genotype, accompanied by lower *in vitro *IL-10 production by stimulated PBMC, and faster melanoma progression after lymph nodes surgery (p = 0.04). With a mean follow-up of 49.5 months post-surgery, one stage IIC patient and 7/8 stage III patients remain NED but 7/7 stage IV patients have progressed.

**Conclusion:**

We conclude that DC/Apo-Nec vaccine is safe, well tolerated and it may induce specific immunity against melanoma Ags. Patients with a low-producing IL-10 polymorphism appear to have a worst prognosis.

**Trial registration:**

Clinicaltrials.gov (NHI) NCT00515983

## Background

Melanoma incidence is constantly rising, but little progress has been made in its treatment. Clinical outcome is highly variable, and prognosis is poor when distant metastases are present [1]. Among the current therapeutic strategies, high dose IFN alpha in stage III patients increases disease-free survival but not overall survival [2], and chemotherapy is seldom useful to control disease progression. Since there is increasing evidence indicating a central role for T lymphocytes in effective immune responses against cancer, considerable efforts are being focused in the field of cancer immunotherapy [3,4]. Dendritic cells (DCs) are professional antigen-presenting cells (APC) that can initiate and regulate T-cell responses by their extraordinary capacity to stimulate naïve T lymphocytes [5,6]. A large variety of antigens (Ags) have been described in melanoma, such as Melan A/MART-1 [7], gp100 [8], MAGE-3 [9], Tyrosinase [10], TRP-2 [11], GD2, GD3 [12] and NY-ESO-1 [13]. Diverse strategies have been reported to generate and assay tumor-specific DC vaccines, which vary in the method of loading Ags in the DCs, in the administration route of the vaccines and in the clinical stage of the patients treated. A randomized trial of autologous peptide-pulsed DCs as vaccines versus DTIC in stage IV melanoma patients did not reveal any advantage between groups [14]. The use of whole tumor cells as a source of Ags has the theoretical potential to elicit a broader immune response to tumor-associated Ags than it would be achieved by pulsing DCs with defined tumor Ags. Thus, Salcedo et al [15] have performed a Phase I/II study on melanoma patients using as vaccines blood monocyte-derived immature DCs (iDCs) loaded with a lysate of a melanoma cell line; they obtained a complete response in 1/15 patients and strong immune specific response against melanoma Ags in 1/5 HLA*0201 patients. Another recent clinical study on 20 melanoma patients demonstrated that *ex vivo *generated DCs loaded with a killed melanoma cell line also induced two clinical responses, one complete and one partial [16]. The low rates of clinical responses obtained, although they constitute a proof of principle, clearly suggest that this type of vaccines should be ameliorated and that other factors, such as the tumor burden and the intrinsic capacity of the patient to react to immunization, should be also considered to evaluate responses. Previous results from our laboratory demonstrated that immunizing mice with iDCs loaded with melanoma B16 apoptotic/necrotic cells efficiently primed both CD4^+ ^and CD8^+ ^T cells and protected 80% of mice against intracutaneous challenge with live B16 cells. This immunization procedure also induced a tumor-specific memory response protecting the animals against a second challenge 10 weeks after vaccination [17]. We and others [18-21] have shown that DC phagocytosis of apoptotic and necrotic tumor cells allows cross-presentation of tumor Ags. Here we describe a human DC-vaccine named DC/Apo-Nec composed of autologous *ex vivo *generated DCs that have phagocytosed an apoptotic/necrotic mixture of four allogeneic melanoma cell lines [21] expressing known melanoma-associated Ags. DC/Apo-Nec cells efficiently cross – presented gp100 and Melan A/MART-1 Ags to induce IFN-γ secretion by specific CTL clones [21]. As previously discussed, melanoma patients respond quite differently to the same vaccination protocol. Since cytokines play a crucial role in the host's immune response, and in view of recent reports that associate melanoma patients outcome with some cytokine gene polymorphisms [22-24], we investigated preliminarily if IL-10 -1082 promoter genotype, which influences cytokine levels in vitro [25], could be related to the overall evolution of the patients participating in the study. Although IL-10 is mainly regarded as an immunosuppressive cytokine, preclinical models as well as observations in humans suggest that it might have a facilitator role in preconditioning tumors to immune recognition [26]. Also, a growing number of reports have suggested an antiangiogenic role for IL-10 [27,28].

In this study, we present the results of a Phase I Clinical Trial of DC/Apo-Nec vaccine in 16 melanoma patients stages IIC, III and IV. We have evaluated the toxicity of DC/Apo-Nec vaccine, the phenotypic characteristics of DCs from treated patients and investigated the T cell responses to Melan A/MART-1 and gp100 Ags in HLA-A*0201 patients by ELISpot and tetramer analysis. The presence of the IL-10 promoter -1082 AA polymorphism in this group of patients appears related to a worst evolution of the disease.

## Methods

### Study design and eligibility criteria

This study was designed as a Phase I clinical trial to evaluate toxicity, feasibility and immune responses to vaccination, and received approval from the Institutional Review Board of the Instituto Alexander Fleming, from an independent Ethics Committee and from the ANMAT, Ministry of Health (Argentina). Eligibility criteria for patients were: (a) histologically confirmed cutaneous melanoma stages IIB, IIC, III or IV (AJCC); (b) patients with minimal or non-detectable disease (NED) after surgery as asserted by CAT scans. Melanoma patients with unknown primary tumor site could be included in the study; (c) ages between 15 and 60 years; (d) life expectancy > 6 months; (e) performance status (ECOG) 0 or 1; (f) patients with stage III disease had to be previously treated with IFN-alpha, and either finished the treatment or suspended it due to disease progression, toxicity or other clinical reasons. Alternatively, patients who had not started IFN-alpha within six months after surgery could be included in this study; (g) a suitable venous access for the leukapheresis procedure; (h) laboratory eligibility criteria included: hemoglobin > 10 gr %; WBC count > 4800/mm^3^, platelets > 150.000/mm^3^, total and direct billirubin, serum oxalacetic transaminase and glutamic pyruvic transaminase < 1.5 fold the upper normal value; LDH ≤ 450 mU/ml; i) absence of pregnancy, with serum βHCG determined one week before vaccination in pre-menopausal women; (i) serum creatinine < 1.4 mg %; (k) no chemotherapy, radiotherapy or any biological treatments during the previous month; (k) no concurrent medication with corticosteroids or NSAIDs; (l) no active brain metastases; (m) normal ECG; (n) all patients gave written informed consent before inclusion in the Study.

### Evaluation of patients and treatment schedule

The baseline tumor evaluation was performed within 35 days prior to the first vaccination. Clinical evaluation included a complete medical history; physical examination; ECG; complete CAT scans (brain, thorax, abdomen and pelvis) to determine tumor staging, tumor burden and sites of disease; blood chemistry and hematology. Physical examination was also performed at the time of each vaccination. Eligible patients underwent on day -30 a leukapheresis procedure to obtain PBMC (peripheral blood mononuclear cells) to generate DCs. Patients received four vaccinations at 2-week intervals, starting at day 0. Vaccination consisted in the intradermal injection of the corresponding vaccine dose (5–20 × 10^6 ^DC/Apo-Nec cells in 300 μl sterile medium) and a DTH reaction in the forearm with 2 × 10^6^irradiated apoptotic-necrotic tumor cells in 100 μl sterile medium without adjuvant. Patients' vital signs and skin reactions were monitored 2 hs after vaccination and DTH was also evaluated 24 and 48 hs post-vaccination. At day 56, the patients underwent laboratory analysis and 40 ml blood extraction to isolate PBMC and serum. At day 70^th^, the patients' status were investigated with abdominal echography and chest X-rays and at day 75^th ^the study ended with a clinical examination.

### Evaluation criteria and statistical analysis

The evaluable population was composed of patients who received the four vaccinations. Because most data groups were not normally distributed, all data were analyzed by the Wilcoxon's rank sum test. All adverse events were classified according to the NCI-Common Toxicity Criteria. DTH scores after the first vaccine (baseline) and after the other three vaccinations were compared between each other by the Mann-Whitney test. For migration experiments and comparison of mean fluorescence intensities of DCs markers expression, statistical analysis was performed by Student's t Test. *P *< 0.05 was considered significant.

### Preparation of tumor apoptotic-necrotic cells

Apoptotic-necrotic (Apo-Nec) tumor cells were prepared as a batch of four cell lines (MEL-XY1; MEL-XY2; MEL-XY3 and MEL-XX4) which have been previously described [21] and were cultured from master cell banks after safety testing for mycoplasma, viruses and bacteria. Cells were cultured in melanoma medium (Dulbecco's Modified Eagle Medium: nutrient Mixture F12 (1:1) (DMEM-F12) supplemented with 2 mM glutamine, 20 nM sodium selenite, 100 μM ascorbic acid, 0.3 mg/ml galactose, 0.15 mg/ml sodium pyruvate and 5 μg/ml insulin, 100 IU/ml penicillin, 10 μg/ml streptomycin) plus 10% fetal bovine serum (FBS) (Natocor, Córdoba, Argentina) in a Good Manufacturing Practice (GMP) core facility at the Centro de Investigaciones Oncológicas-FUCA. After gamma irradiation at 70 Gy (Siemens Lineal Accelerator), the cells were frozen in freeze-medium (50% DMEM, 40% human albumin and 10% DMSO) in liquid nitrogen until use. A soft agar clonogenic assay performed in sextuplicate (10^4 ^seeded cells/well) was used to test that irradiated cells have lost their proliferation ability compared to non-irradiated control cells [29]. To prepare each vaccine, the cells were thawed, washed and plated for 72 hs to complete the apoptotic process (Apo-Nec cells). After that, the cells were detached from the flasks, counted and resuspended in AIM-V Medium (Therapeutic grade, GIBCO, Invitrogen Corporation, Grand Island, N.Y).

### DCs generation, characterization and vaccine preparation

DCs were generated at the Centro de Investigaciones Oncológicas-FUCA according to GMP. PBMC were obtained with a Continuous Flow Cell Separator (Fressenius AS 104) using a modified protocol for granulocytes isolation and a simple stage P1Y equipment. A peripheral venous double punction without previous patient stimulation was performed. Average values of initial hemograms were: 6.8 × 10^3 ^per mm^3 ^WBC (range 4.7–9.7 × 10^3^/mm^3^) and 29.5% lymphocytes (range 16.3–43%). An average blood volume of 6 liters was processed (range 5–7 liters), with a mean product volume of 140.7 ml (range 99–250 ml). Mean PBMC yield was 7.5 × 10^9 ^cells (range 3.0–11.3 × 10^9^). Whole procedure time was 90 min, and no adverse events were observed. PBMC were purified by Ficoll-Hypaque gradient centrifugation and frozen in freeze-medium on day -30. To prepare each vaccine, mononuclear cells were thawed one week before and monocytes were allowed to adhere to plastic for 2 hs in AIM-V Medium in 0.22 μm filter-capped culture flasks. After removing the lymphocytes, immature DCs (iDCs) were obtained by culturing for five days in AIM-V medium in presence of 800 U/ml GM-CSF (rhGM-CSF; Molgramostim, a generous gift from Dr. Esteban Corley, PC Gene, Buenos Aires, Argentina) and 50 ng/ml IL-4 (rhIL-4, Peprotech, Mexico). According to the vaccine dose, 5, 10, 15 or 20 × 10^6 ^DCs were cocultured with Apo-Nec cells in a 3:1 as previously reported [21] in AIM-V medium for 48 hs at 37°C. On the vaccination day, cocultures were centrifuged at 1200 rpm for 5 min, gently suspended in 300 μl and injected *i.d*. in an arm or thigh with intact draining lymph nodes.

For each patient, quality controls (viability, cell count and purity) were performed on all DCs preparations. Sterility testing was performed on the starting leukapheresis product, Apo-Nec cells and on the final coculture. DC preparations were characterized by flow cytometry (FACS) in a FACScalibur (BD Biosciences, San José, CA, USA). The following mAbs were used: CD1a, CD11c, CD14, CD80, CD83, CD86, HLA-DR, CD40, HLA ABC, CCR7 or the corresponding isotype-matched controls, all from BD Biosciences (San José, CA, USA).

DCs endocytosis was evaluated by incubating 10^6 ^cells with 1 mg/ml FITC-dextran (FITC-Dx) (Sigma, St Louis, CA) for 60 min at 37°C. Controls included tubes incubated with FITC-Dx at 4°C to inhibit the endocytic process and a basal uptake performed at 0 time point. After washing with Phosphate Buffered Saline (PBS), FITC-Dx uptake was quantified by FACS analysis (10,000 cells per point). DCs migration was assessed *in vitro *before and after co-culture with Apo-Nec cells, using a 48-wells chemotaxis chamber (AP 48 Neuroprobe Inc., Gaithersburg, MD) as previously described [21]. iDCs cultured in the presence of 2 μg/ml lipopolysaccharide (LPS) for 48 hs were used as a maturation control for every DC experiment.

### Samples for immunological tests

To assess the humoral immune response, pre-vaccination sera were obtained on day -7th and post-vaccination sera on day 56th (fourteen days after the last vaccination), aliquoted and stored at -80°C. PBMC were purified by Ficoll-Hypaque gradient centrifugation from the leukapheresis product (pre-vaccination PBMC) or from 40 ml of heparinized blood obtained two weeks after the last vaccination (post-vaccination PBMC) and frozen in freeze-medium until testing. HLA typing for HLA-A*0201 was determined after incubation of patients' PBMC samples with mouse monoclonal anti-HLA-A2 FITC conjugated (BD-Pharmingen, San Jose, CA).

### DTH reactions

On each vaccination day, DTH was performed in the forearm with 2 × 10^6 ^Apo-Nec cells and the reaction was read at 2, 24 and 48 hs. A DTH intensity value was established as follows: 0: erythema < 0.5 cm diameter; 1: macular erythema 0.5–1.0 cm; 2: macular erythema 1.0–2.0 cm; 3: macular erythema > 2.0 cm or papular erythema<1.5 cm;4: papular erythema > 1.5 cm. The DTH score corresponds to Σ all individual DTH intensities/4. DTH measurements were not blinded.

### Lymphocyte proliferation assay

In the case of patient #1 we could establish a melanoma cell line from an axillary lymph node metastasis resected before entry in this clinical study. Apo-Nec cells from patient # 1's tumor cells (Apo-Nec#1) were obtained by gamma irradiation as described above. Patient #1's DCs cocultured with Apo-Nec # 1 cells (1.5 × 10^4 ^DCs + 5 × 10^3 ^Apo-Nec # 1) for 48 hs were plated in 96-well U-bottom plates in triplicate and pre- and post-vaccination PBMC from patient #1 (2 × 10^5^) were thawed and added to DC/Apo-Nec # 1. Controls were performed with PBMCs alone or PBMCs plus Apo-Nec # 1 cells. As a positive control, pre- and post-vaccination PBMC were treated with 5 μg/ml phytohemagglutinin A (PHA, Gibco, Grand Island, NY). After 96 hs of culture, cells were pulsed with [H]^3 ^dThd (Amersham, 1 μCi/well) during the last 16 hs. The cells were lysed with a Cell Harvester (Nunc, Rochester, NY) and radioactivity was measured in a Liquid Scintillation Counter. The experiments were performed in triplicate and the mean ± SD is shown.

### Immunomonitoring

#### Staining with HLA/peptide tetramers

Phycoerythrin or allophycocyanin-conjugated HLA-A*0201 tetramers (AAGIGILTV or KTWGQYWQV, respectively) were kindly supplied by the NIH Tetramer Core Facility (Emory University, Atlanta GA) and used to identify specific clones for Melan A/MART-1 or gp100 respectively. Pre- and post-vaccination PBMC samples from HLA-A*0201 patients were thawed and incubated for 2 hs at 37°C in AIM-V medium, stained with tetramers at 37°C for 15 min and immediately chilled on ice. Samples were then incubated with anti-CD8 FITC (BD Biosciences, San Jose CA) at 4°C for additional 40 min, washed with PBS and analyzed by FACS. As positive controls we used Melan A/MART-1 (M27) or gp100 (G154) specific HLA A*0201 restricted CTL clones [30] expanded in RPMI medium in 14-day cycles in the presence of 30 ng/ml anti-CD3 antibody (OKT-3, BD Biosciences) and serial 300 UI/ml IL-2 (Chiron BV) every 3 days plus 10% heat-inactivated AB human serum and antibiotics. Negative controls were performed with PBMC samples from healthy HLA-A*0201 donors.

#### ELISpot assay for IFN-γ release from single Ag-specific T cells

In HLA-A*0201 patients, stimulator DCs were prepared from CD14^+ ^cells purified from PBMC using CD14 MicroBeads and autoMACS separator (Miltenyi Biotec, Paris, France), cultured for 5 days in synthetic medium SYN-H (AbCys, Paris, France) containing 100 ng/ml rhGM-CSF and 20 ng/ml IL-4, and maturated with 10 μg/ml LPS for 2 additional days. Mature DCs were pulsed for 1 h at 37°C with 10 μg/ml peptide diluted in SYN-H medium, washed and used as stimulators for CD8+ T cells. We used gp100 (KTWGQYWQV), MelanA/MART-1 (AAGIGILTV) and Flu_58–66 _(GILGFVFTL) (influenza virus) HLA A*0201 restricted peptides. Nitrocellulose plates (96-wells; MAIPS 450; Millipore, Bedford, MA) were coated overnight at 4°C with 10 μg/ml anti-human IFN-γ mAb (Mabtech, Nacka, Sweden) in 50 mM carbonate-bicarbonate buffer pH 9.6, washed and blocked with IMDM (Iscove Modified Dulbecco's Medium, Gibco-Invitrogen, Cergy Pontoise, France)/10% human AB serum (Biowest, Nuaille, France) for 1 h at 37°C. PBMC were stained with anti-CD8 PC5 (Beckman Coulter) and counted by FACS analysis. Effector cells (E) (10^5 ^CD8^+ ^T cells/well) were seeded in 100 μL medium and stimulator cells (S) added to reach an E/S ratio of 10: 1 in a total of 200 μL/well. After 24 hs incubation, the supernatants were separated and wells were washed 5 times with 0.1% Tween-20 in PBS. Plates were incubated 2 hs at room temperature (RT) with 1 μg/ml biotinylated mouse anti-human IFN-γ mAb (Mabtech) in PBS/HSA (human serum albumin) (0.4 g/L). After extensive washing with 0.1% Tween-20 in PBS, 1: 1000 streptavidin-alkaline phosphatase (Mabtech) in PBS/HSA was added and incubated for 1 h at RT. The plates were then washed and incubated with 5-bromo-4-chloro-3-indolyl phosphate/nitroblue tetrazolium substrate for 30 min at RT (Mabtech). Color developing was stopped by washing under running tap water. After drying, IFN-γ secreting T cells were counted using an automated image analysis system ELISpot reader (AID, Strassberg, Germany). Positive controls were done with DCs pulsed with Flu_58–66 _peptide. Negative controls were either non-pulsed DCs or DCs pulsed with CMV_495–503 _(NLVPMVATV) peptide. Each experiment was performed in triplicate.

### Determination of humoral responses

#### Indirect Immunofluorescence

The presence of anti-melanoma antibodies was investigated by incubation of live melanoma cell lines that comprise Apo-Nec vaccine (mixed in equal proportions) with pre- and post- vaccination sera for 1 h and detected as previously described [31].

#### Western Blots

Protein extracts were prepared from the four cell lines that comprise Apo-Nec vaccine by lysis with 1% NP40, homogeneized with a Polytron (Brinkmann Instruments, USA) and centrifuged for 40 min at 10.000 ×g. The protein concentration was measured according to Lowry [32]. Protein extracts were prepared equally from the human breast cancer cell line IIB-BR-G [33] and used to assess specificity in the Western Blots. Western blots were performed as described [31] using pre- and post-vaccination sera as primary antibodies.

### IL-10 promotor polymorphism analysis

DNA was extracted from heparinized blood samples, using CTAB extraction protocol [34]. PCR for genotyping IL-10 -1082 was performed using primers and amplification conditions described by Marka et al [35]. The reverse primer contained an additional Mnl I restriction site within the primer recognition sequence. After amplification, PCR products were purified from an agarose gel with a home-made glass fiber column. RFLP was performed by overnight digestion with Mnl I restriction endonuclease (New England Biolabs, Beverly, USA). The products were analyzed after electrophoresis on 3% agarose gel and ethidium bromide staining (Gibco, BRL). Three different genotypes have been associated to different *in vitro *IL-10 production: GG (high production), AG (moderate production) and AA (low production). Results of the RFLP were interpreted as follows: 94, 65 and 39 bp bands correspond to GG genotype; 133, 94, 65 and 39 bp bands correspond to AG genotype and 133 and 65 bp bands correspond to AA genotype.

### IL-10 measured by ELISA

IL-10 concentrations were determined in the sera of vaccinated patients before (pre-vaccination serum) and three weeks after the fourth vaccination (post-vaccination serum). Sera were frozen at -80°C until tested by ELISA in triplicate (OptEIA IL-10, BD Biosciences, San Diego, CA).

To measure *in vitro *IL-10 production by patient's PBMC in response to LPS or PHA stimulation, 1 × 10^6 ^cells were thawed, plated in 2 ml AIMV medium in 35 mm petri dishes for 24 hs, and were either untreated or treated with 10 μg/ml LPS or 20 μg/ml PHA for another 24 hs [36]. Culture supernatants were collected, centrifuged at 2000 rpm and tested by ELISA as indicated above. A calibration curve was performed for each experiment and the sample concentration was calculated by log-log regression analysis using Cembal 2.2 software.

## Results

### Patients

This Phase I Study was performed between October 2004 and September 2005, on sixteen melanoma patients from the Instituto Alexander Fleming. Their characteristics are listed in Table 1. The mean age was 42 years (range 17–60 years-old). There were five females and eleven males. One patient had AJCC stage IIC melanoma, eight had stage III melanoma and seven had stage IV melanoma (five M1b and two M1c). Seven of eight Stage III patients received IFN-α treatment before entering this study: two completed their treatment; four interrupted it due to progressive disease, and one due to hepatic toxicity. Cohorts of four patients were vaccinated with 5, 10, 15 or 20 × 10^6 ^DCs cocultured with Apo-Nec cells (DC/Apo-Nec vaccine). Every patient received one dose of DC/Apo-Nec (0.3 ml) without adjuvant every two weeks. Patient #7 was withdrawn from the protocol after the second vaccination due to rapidly progressing disease after a sport trauma in his right thigh and uncontrolled infection; this patient was not replaced.

**Table 1:** Characteristics of the patients participating in the Clinical Study

**Pt #**	**Sex**	**Age**	**Stage**	**Mts Site**	**MN Cells obtained (×10^9^cells)**	**Dose of DC/Apo-Nec (×10^6^cells)**	**No. Vacc**	**Clin. Evolution (12/1/07)**	**IL-10 Promoter Genotype**
**1**	F	42	III	LN	3.5	5	4	P (10 m)	AA
**2**	F	57	III	ND	3.6	5	4	NED (62 m+)	AG
**3**	M	32	III	ND	3.3	5	4	NED (43 m+)	AA
**4**	F	17	III	ND	4.4	5	4	NED (53 m+)	AG
**5**	M	56	IV	L,SC	5	10	4	P (4 m)	AA
**6**	M	60	III	ND	1.5	3	4	NED (45 m+)	AA
**7**	M	27	IV	SC	4.2	10	2	WP (1 m)	AA
**8**	M	26	IV	ND	3.6	10	4	P (7 m)	AA
**9**	F	42	III	ND	7.5	15	4	NED (79m+)	AG
**10**	M	34	IV	LN	6.2	15	4	P (11 m)	AG
**11**	M	44	IV	L	4.7	15	4	P (4 m)	AG
**12**	M	56	III	ND	8	15	4	NED (33m+)	AG
**13**	M	47	IIC	ND	9.3	20	4	NED (34 m+)	AA
**14**	M	30	III	ND	7.5	20	4	NED (47 m+)	GG
**15**	M	52	IV	LN	6	20	4	P (10 m)	AA
**16**	F	57	IV	SC	8.2	20	4	P(6 m)	AA

ND: non-detectable disease after surgery; SC: subcutaneous; L: Lung; LN: Lymph node; NED: non-evident disease; P: progression; D: deceased. WP: withdrawn from protocol. m: months of follow-up from last surgery.

### Characteristics of DC/Apo-Nec vaccine obtained for melanoma patients

**Figure 1:**
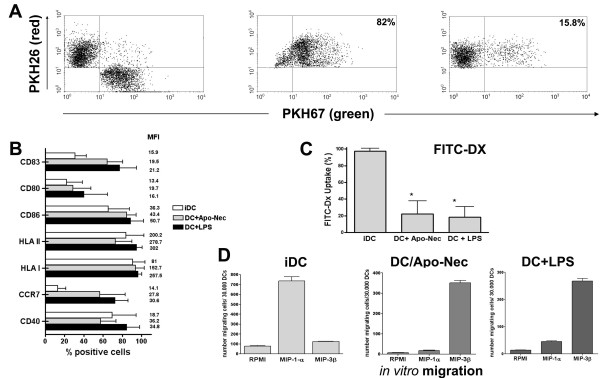
**Characteristics of DC/Apo-Nec vaccine**. **A-DCs phagocytosis**. Patient #1 results are shown. Apo-Nec cells (green labeled) phagocytosis by DCs (red-labeled, left panel) was evaluated as described under Methods. DCs and Apo-Nec cells were co-cultured for 48 hs at 37°C (middle panel) or at 4°C to inhibit phagocytosis (right panel). Total DCs (red labeled population) was gated and the percentage of double positive cells (DC/Apo-Nec) was calculated. **B-DC Maturation**. Markers expression of iDCs, DC/Apo Nec cells and DCs + LPS from all vaccinated patients (n = 15) was evaluated by FACS using monoclonal antibodies as described under methods. Results are mean ± SD percentage of positive cells, MFI: mean fluorescence intensities. **C- DC endocytosis**. FITC-Dx uptake of iDCs DC/Apo-Nec cells and DC + LPS of all vaccinated patients (n = 15). Results are indicated as mean ± SD percentage of FITC-Dx positive cells after 90 min incubation and washing. *: diferences with iDCs were statistically significant (Student's t test). **D-DC migration**. *In vitro *migration towards MIP-1α and MIP-3β was measured as described under methods. iDCs, DC/Apo-Nec and DCs + LPS from patient # 9 are shown (mean ± SD, assayed in triplicate).

For each vaccination, DCs were generated from a frozen aliquot of PBMC after culture of adherent monocytes in serum-free medium in the presence of rhGM-CSF and rhIL-4. At day 5, immature DCs (iDCs), were obtained which were CD14^- ^/CD11c^+ ^(95.1 ± 3.6%) and CD1a^+ ^(70 ± 9%, data not shown). Approximately 3 × 10^6 ^DCs were obtained from 1 × 10^8 ^PBMC plated in serum-free medium. At day 5, iDCs were co-cultured with Apo-Nec cells, incubated for 48 hs, resuspended as described under Methods and injected into patients. In Figure 1 we summarize the characteristics of iDCs and DC/Apo-Nec vaccine obtained for the melanoma patients participating in the study. We found that patient's iDCs were able to phagocytose Apo-Nec cells (42.3 ± 13.7%, PKH 26/PKH67 double labeled population, n = 15), and in Figure 1A patient # 1 DCs phagocytosis of Apo-Nec cells is shown as an example. The ability of Apo-Nec cells to influence the maturation process of monocyte-derived DCs was examined by means of FACS measurement of specific DC markers. Phagocytosis of Apo-Nec cells yielded a mature-DC phenotype (Figure 1B) as compared to a LPS-incubated control. DCs maturation was evidenced by increased expression of CD83, CD80, CD86, HLA class I and II and CD40. Also, after phagocytosis, a 75.2% ± 16 reduction in FITC-Dx endocytosis was observed compared to iDCs (Figure 1C).

Chemokine (C-C motif) receptor 7 (CCR7) was clearly upregulated upon Apo-Nec phagocytosis in DCs from all patients (Figure 1B) and this correlated with DCs migration towards MIP-3 beta *in vitro*. As an example, we show in Figure 1D patient # 9 DCs migration towards MIP-1α and MIP-3β. Immature DCs (9.6% CCR7+, MFI 23.3) migrated to MIP-1 α but failed to respond to MIP-3β; instead DC/Apo-Nec cells (81.8% CCR7+, MFI: 41.2) clearly increased their migration to MIP-3-β but minimally responded to MIP-1α.

### Toxicity to DC/Apo-Nec Vaccine

Except for patient # 6, in whom the yield of PBMC was low, and therefore the expected dose of 10 × 106 DC/Apo-Nec could not be attained (he only received 3 × 106 DC/Apo-Nec per vaccine), all other patients received the expected dose of DC/Apo-Nec. DC/Apo-Nec vaccine was well tolerated and only mild toxicity, always Grade 1, was found. Weak local reactions at the vaccination and DTH sites, consisting in erythema and papulae, were observed. No clinical manifestations of autoimmune disease developed in any patient (Table 2).

**Table 2:** Toxicities associated with DC/Apo-Nec vaccination (*)

**Symptoms**	**DC/Apo-Nec 5 × 10^6^**	**DC/Apo-Nec 10 × 10^6^**	**DC/Apo-Nec 15 × 10^6^**	**DC/Apo-Nec 20 × 10^6^**
**Fatigue**	1/4	1/3	0/4	0/4
**Headache**	1/4	0/3	0/4	0/4
**Chills**	1/4	0/3	0/4	0/4
**Abdominal cramps**	0/4	0/3	0/4	1/4
**Local reaction**	4/4	3/3	4/4	4/4
**Astenia**	0/4	1/3	0/4	0/4
**Nausea**	0/4	0/3	0/4	1/4
**Abdominal pain**	0/4	0/3	0/4	1/4
**Vomiting**	0/4	0/3	0/4	1/4
**Anorexia**	0/4	1/3	0/4	0/4
**Diarrea**	0/4	0/3	0/4	1/4
**Myalgia**	1/4	0/3	1/4	0/4

(*) Toxicity was Grade 1 in all cases.

### Clinical evolution in vaccinated patients

As of December 2007, with a mean follow-up of 49.5 months post-surgery (33–79 months), the stage IIC patient is NED; 7/8 (87.5%) stage III patients are NED and 7/7 stage IV patients experienced disease progression. No regressions of metastatic lesions were observed in this study.

### Immune responses

#### DTH

DTH reactions to Apo-Nec cells were measured at each vaccination. The intensity of the reaction and the DTH score were determined as described under Methods. Only few patients (6/15) had a weak pre-vaccination DTH reaction against Apo-Nec cells. DTH test revealed vaccination – induced Apo-Nec cells specific reactivity in all patients, since DTH score was significantly higher after the second DC/Apo-Nec vaccine compared to the basal reaction observed after the first-vaccination (Mann Whitney test P = 0.0039, n = 15) (data not shown). Although without reaching statistical significance, DTH scores were higher in NED patients than in those who experienced disease progression. The increase in DC/Apo-Nec number per vaccination did not increase the DTH score significantly (not shown).

Patient #1 had a DTH response to her autologous Apo-Nec cells after vaccination, demonstrating that vaccination with allogeneic DC/Apo-Nec cells induced immunity against her own melanoma Ags (not shown).

#### Humoral response

No humoral responses were detected against the live melanoma cells that comprise the vaccine, either before or after vaccination, as determined by FACS analysis. We also investigated the presence of anti- Apo-Nec cells antibodies in pre- and post- vaccination sera by Western blot. In four patients (#3, #4, # and #16) we observed only a faint protein band (>200 kDa) in post-vaccination sera which was not present in breast cancer cells extract used as unspecific control (not shown).

### Lymphocyte proliferation in response to autologous tumor cells (patient # 1)

**Figure 2:**
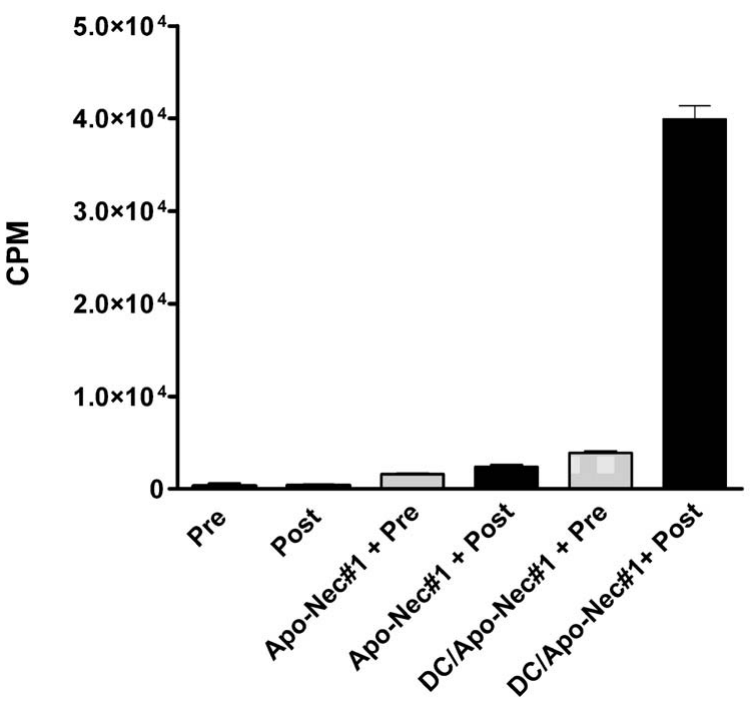
**Patient#1's lymphocytes in vitro proliferation in response to autologous tumor Ags presented by DCs**. Pre and post vaccination Patient#1 PBMC were incubated either alone, with Apo-Nec#1 cells prepared from patient#1 tumor cells or with DC/Apo-Nec#1 cells; (H^3^) dThd incorporation was measured as described under Methods. Results represent mean ± SD cpm (counts per minute) of triplicates. Positive controls incubated with PHA incorporated more than 7 × 10^4 ^cpm (not shown).

In the case of patient #1, an autologous melanoma cell line was established, allowing us to analyze if lymphocytes were primed by DC/Apo-Nec vaccination (allogeneic cells) and recognized autologous Ags. We measured pre- and post-vaccination PBMC proliferation after a five-day coculture of mononuclear cells with Apo-Nec#1 cells (irradiated patient #1's tumor cells) or DC/Apo-Nec as described under Methods. As we had previously demonstrated, Apo-Nec cells alone were not able to stimulate PBMC by their own, probably due to the loss of HLA-class I molecules at the cell surface during the apoptotic process [21] (Figure 2). Instead, post-vaccination PBMC proliferated approximately 10-fold more than pre-vaccination lymphocytes in response to DC/Apo-Nec#1 cells, demonstrating that after vaccination with DC/Apo-Nec, lymphocyte clones recognizing Ags shared by the Apo-Nec mixture and the patient's tumor cells had expanded.

### Tetramer analysis and IFN-γ- ELISpot for gp100 and Melan A/MART-1

7/15 patients enrolled in this Phase I Clinical Trial had the HLA-A*0201 class I haplotype, and thus HLA-restricted tumor-specific responses could be studied in their PBMC samples. We analyzed anti-gp100 and Melan A/MART-1 specific CD8+T cell responses induced by DC/Apo-Nec vaccine, by: i) specific HLA/peptide tetramer binding and ii) IFN-γ ELISPot, directly from peripheral blood samples.

**Figure 3:**
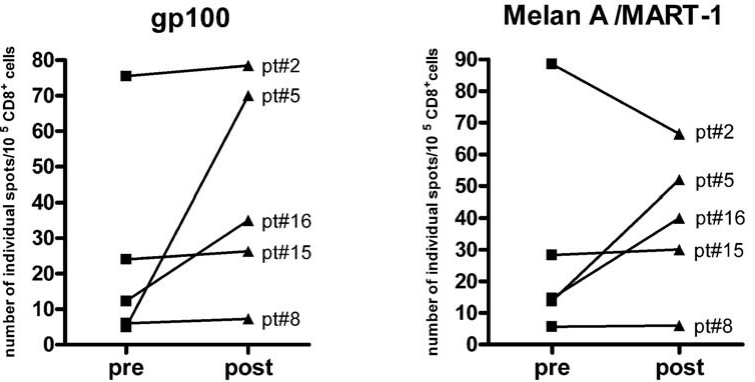
**Detection of antigen-specific IFN-γ secreting CD8+T cells by ELISpot analysis**. ELISpot assay was performed as described under Methods. Number of individual gp100 and Melan A/MART-1 specific spots/10^5 ^CD8+T cells from pre and post DC/Apo-Nec vaccination samples are shown for 5/7 HLA-A*0201 patients. Data represents mean values of triplicates. Background numbers of spots were set with non – pulsed DCs and subtracted from the number of spots.

i) For five patients enough PBMC were obtained before and 14 days after the fourth vaccination to analyze the presence of gp100 and Melan A/MART-1 specific CD8+T cells by tetramer staining. We observed an irregular trend. In some patients, i.e. patients #2 and #6, tetramer positive cells increased several fold to over 1% for both Ags tested after DC/Apo-Nec vaccination. Patient #8 had a high frequency of anti-Melan A/MART-1 reactive CD8+T cells before treatment that did not change after vaccination (pre: 0.98%; post: 0.93%); instead, gp100-specific T cells decreased after vaccination (pre: 0.93%; post: 0.25%). Patient #16 had a high number of pre-vaccination CD8+ gp100- and Melan A/MART-1-specific lymphocytes (1.9 and 1.7%, respectively) which decreased 50% after vaccination (1 and 1.1%, respectively). No staining was observed in an HLA-matched healthy donor under the same conditions (not shown).

ii) As IFN-γ plays a crucial role in the induction of tumor-protecting T cell immunity, it is important to measure the frequency of Ag-specific individual IFN-γ secreting CD8+ T cells in treated patients. Total pre-and post-vaccination PBMC were tested for release of IFN-γ in ELISpot after 24 hs incubation with autologous DCs pulsed with gp100 or Melan A/MART-1 peptides and, as a control, influenza (flu_58–66_) peptides. This assay was evaluated in 5/7 HLA-A*0201 patients as shown in Figure 3. We observed that in two patients (#5 and #16) DC/Apo-Nec vaccination induced an increase of anti- gp100 and MelanA/MART-1 IFN-γ secreting CD8+T cells. For gp100, frequencies of 70 × 10^-5 ^and 35 × 10^-5 ^CD8+ T cells secreting IFN-γ were induced, while pre-vaccination values were negligible. For patient #2 the situation was peculiar since this patient had a high frequency of IFN-γ secreting CD8+ clones specific for gp100 and Melan A/MART-1 even before vaccination, and this high frequency did not change (gp100) or slightly decreased (Melan A/MART-1) after vaccination. Patients #15 and # 8 had intermediate or negligible numbers of spots at baseline (pre-vaccination) and after vaccination. Positive controls performed with Flu_58–66 _peptide detected 15–20 spots in each sample (not shown).

### IL-10 promoter polymorphism

**Figure 4:**
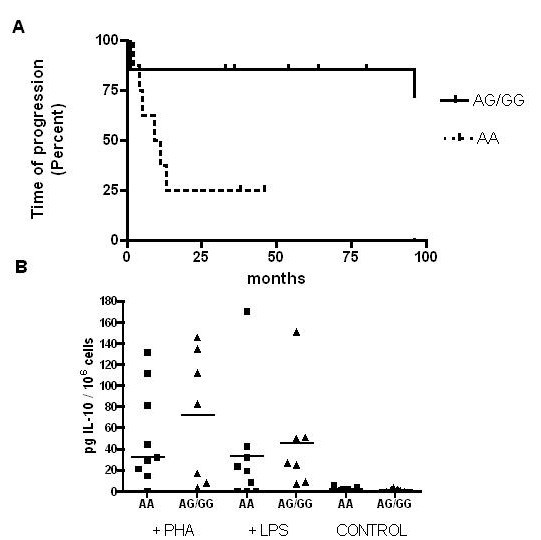
**Influence of Interleukin-10 -1082 promoter genotype in disease progression**. **A- **Disease progression was calculated in months from the last surgery, reported as of December 2007. Differences between curves were significant (P = 0.04, Chi Square test). **B- ***In vitro *IL-10 secretion by patients' PBMC in response to LPS or PHA. Control PBMC (not stimulated) did not secrete IL-10. Culture supernatants were tested in triplicate by ELISA as described under methods. Lines represent median value in each case. Mann Whitney's test showed that differences between AA and AG/GG genotypes were not statistically significant. AA (PHA) vs AG/GG (PHA) P value: 0.6; AA (LPS) vs AG/GG (LPS) P value: 0.25.

Polymorphism in the IL-10 promoter has been associated with differential *in vitro *secretion of IL-10, and various authors have reported that these polymorphisms may influence the outcome of melanoma patients [37,38,22]. Genotyping for -1082 (G/A) includes three categories: GG is associated with high *in vitro *tumor IL-10 production, whereas AG and AA, are associated with intermediate and low production, respectively [22]. As the AA genotype has been shown to be associated with decreased survival in patients with advanced disease [37] we analyzed the -1082 genotype in all patients. In order to choose a common starting point of observation, the influence of IL-10 polymorphism on the interval that elapses between surgery due to lymph node involvement (stage III) and progression to distant metastases (stage IV) was studied. Therefore, patient # 13 was not included in this analysis, since he was at clinical stage IIC. Although numbers are small and should be cautiously interpreted, as observed in Figure 4A patients with AA genotype were associated with more rapid disease progression after lymph node surgery than patients with AG/GG genotypes (P = 0.04, curves compared by Chi Square test). To correlate genotype with the actual IL-10 production by stimulated PBMC, *in vitro *IL-10 secretion by pre-vaccination PBMC from all patients was investigated after LPS or PHA stimulation. As observed in Figure 4B, PBMC from AA patients secreted less IL-10 in response to both stimuli than those from AG/GG patients; although the differences were not statistically significant (Mann Whitney Test), the trend was clear. Control PBMC (not stimulated) secreted less than 7 pg IL 10/10^6 ^/ml (detection level in ELISA).

## Discussion

In the present Phase I study performed on sixteen melanoma patients stages IIC, III and IV, we have assayed a therapeutic DC-based vaccine (DC/Apo-Nec) in which autologous iDCs were loaded with a mixture of apoptotic/necrotic allogeneic melanoma cell lines. Accordingly to our preclinical results obtained with DCs from healthy donors [21], we report here that phagocytosis of Apo-Nec cells induced DCs maturation, a requirement reported as essential to trigger an effective immune response [39,40]. These findings contrast with those of Neves et al [41] who reported hat monocyte-derived DCs from cancer patients mature less efficiently than those from healthy volunteers. We have also previously demonstrated that this strategy allows Ag processing and cross-presentation for CTL priming [21], and therefore permits the "natural" processing and epitope selection of known as well as yet unknown Ags, allowing presentation of a great peptide diversity on various HLA-haplotypes. DC/Apo-Nec vaccine was well tolerated and safe, since only mild toxicity was found, and induced cellular responses, since DTH reactions to the Apo-Nec cells increased in all patients. DTH reactions have been used as an indicative of *in vivo *induction of immunological memory, and some authors have reported a positive correlation between DTH and patients outcome [42,43].

DTH was not exclusively triggered by the allogeneic vaccine, since at least in patient #1 we observed, after vaccination, a positive DTH to the patient's own irradiated tumor cells, demonstrating that DTH also took place when autologous Ags were used. For this patient we also found specific *in vitro *PBMC proliferation to DC/Apo-Nec#1 cells after vaccination. We have also demonstrated by ELISpot analysis that vaccination with DC/Apo-Nec elicited high numbers of anti-MelanA/MART1 and anti-gp100 specific CD8+T lymphocytes in 2/5 analyzed HLA-A*0201 patients; 2/5 patients had invariant high or intermediate levels of specific CD8+T lymphocytes, and 1/5 patient did not develop any specific T cell response. We have also used the tetramer binding analysis to quantify CD8+T cells recognizing the same two Ags. The results observed with this methodology were irregular, with some patients increasing their tetramer-positive PBMC to over 1% for one Ag while decreasing for the other. It should be taken into consideration that the number of circulating PBMC specific for a given Ag reflects a balance between production and migration to peripheral tissues, perhaps where tumor may be present [44]. Several observations suggest that MelanA/MART-1-specific CD8+ T cells can mediate tumor regression. This conclusion is based on the cytolytic activity of MelanA/MART-1-tetramer+ lymphocytes in culture [45], on the poor prognosis for patients with MelanA/MART-1-negative primary melanoma [46] as well as on results of adoptive immunotherapy with autologous MelanA/MART-1-specific CTL clones [47]. Most antitumor vaccination attempts failed to elicit significant specific T cells numbers, which remained either undetectable [48] or in low frequencies in *ex vivo *assays [49]. Besides, T cells need to be in the correct state of activation to perform their functions and mediate effective immune protection against cancer [50]. It is remarkable that, for all analyzed patients, the numbers of CTLp (quantified by ELISpot) were strikingly lower than the number of tetramer-positive CD8+T lymphocytes (data not shown), thus suggesting that despite Ag recognition, their functional properties were impaired. The possibility that not all tetramer-positive cells may be functional has also been demonstrated in EBV, HIV-1 and tumor-associated antigen-specific CD8+T cells [51-53]. Besides, even the induction of high numbers of circulating Ag-specific CD8+T lymphocytes may not be related to clinical benefit since, as recently reported [54], profound differences can be found between tumor-specific CD8+ T cells in circulation and those isolated and/or studied in the tumor microenvironment, where the presence of high levels of FoxP3+ regulatory T cells could influence their functional status. These evidences, among others [55], suggest that a strong phenomenon of immune tolerance takes place within the tumor including the presence of regulatory T cells, thus hampering effective T cell functionality. This effect is probably more important as the tumor mass increases, and less so when tumor cells are fewer in number, as in the adjuvant setting.

Recent reports tried to elucidate if certain polymorphisms that result in functional changes in cytokine genes could influence susceptibility, disease progression or survival in cancer patients. In melanoma, the AA genotype has been associated to a poorer prognosis, supporting the recent findings that IL-10 has an anti-tumor effect possibly via inhibition of angiogenesis [27,28]. The presence of the AA genotype (lower IL-10 production) has also been associated to the development of prostate cancer and renal cell carcinoma [56,57] but not conclusively to melanoma susceptibility [24,58]. Also, a recent report [22] has suggested that polymorphisms associated with low expression of anti-inflammatory IL-10 and TGF-β1 and immunomodulatory TNF-α, IFN-γ and IL-6 could be involved in the mechanisms of cancer progression and escape from immune surveillance. A dual role for IL-10 in the tumor microenvironment has been hypothesized: IL-10 is secreted constitutively by some tumors where it would have two major roles (1) to increase NK cell tumor killing, release of Ags and possibly APC uptake of damaged tumor cells and (2) to inhibit APC maturation with consequent maintenance of the ability to uptake Ags, remaining *in situ *rather than migrating to regional lymph nodes. Thus, IL-10 would maintain a chronic inflammatory state within the tumor microenvironment and it would also inhibit recruitment and activation of adaptative immune system [26].

We analyzed if the IL-10 -1082 promoter polymorphism in our group of patients could be related to the rate of disease progression from stage III to stage IV. Although the number of patients was small, we found a significant correlation between AA genotype and rate of disease progression. The genotype and phenotype were correlated, since after measuring *in vitro *IL-10 secretion of patients' PBMC treated with LPS or PHA. AA patients showed a trend to lower IL-10 production than AG/GG patients, although the difference was not statistically significant probably due to the small number of patients analyzed. Thus, our results suggest that AA patients (low IL-10 *in vitro *production) had a worse outcome than the intermediate/high IL-10 producing patients. In this study, the number of patients was too small to analyze the correlation between IL-10 genotype and response to vaccine, since 5/7 (71%) stage IV patients had the most unfavorable genotype (AA) versus 3/8 (37%) for stage III patients.

Although we did not observe clinical responses in metastatic melanoma patients, 87.5% of the stage III patients are disease-free with a mean follow up of 49.5 months after surgery. Thus, we believe that this vaccination approach could benefit patients in the adjuvant setting since the limited number of activated Ag-specific CD8+ lymphocytes would be only able to eliminate micrometastatic lesions, thus protecting patients from tumor relapse.

## Conclusion

Our results demonstrate that the use of *ex vivo *generated DCs loaded with a mixture of allogeneic melanoma cell lines is safe and it elicits immune response against tumor Ags, shown by: i) an increased DTH; ii) an increase of anti-Melan A/MART-1 and anti-gp100 lymphocytes; iii) activation of lymphocytes directed to as yet unspecified Ags. An association of IL-10-1082 AA promoter polymorphism with worst prognosis in melanoma is suggested and should be further investigated. Larger numbers of patients should be treated with DC/Apo-Nec vaccine to evaluate its clinical efficacy in a Phase II clinical trial.

## Competing interests

The author(s) declare that they have no competing interests.

## Authors' contributions

EMvE and MMB contributed equally to this work. They have prepared DC/Apo-Nec vaccines, performed DCs characterization (maturation studies), migration experiments, cross-presentation studies, designed experiments to measure immune response and drafted the manuscript.

DF and IP performed ELISpot determinations.

EML performed IL-10 promoter polymorphism characterization.

MB helped with figures preparation.

OL contributed to design ELISpot assays.

AV and AK performed leukapheresis procedures and set up optimal conditions to enrich samples in monocytes fraction.

CY provided the CTL clones for assessment of DC/Apo-Nec cross-presentation.

RW participated in the discussion of the results.

JM was the Responsible Investigator of the Study, participated in its design and coordination and also helped to draft the manuscript.

All authors read and approved the final manuscript.
